# The Effect of Preoperative Visual Explanation on Anxiety in Children: A Randomized Controlled Trial

**DOI:** 10.3390/jcm15031041

**Published:** 2026-01-28

**Authors:** Hülya Tosun Söner, Süleyman Kızıldağ, Osman Uzundere, Fatma Acil, Meral Erdal Erbatur, Selen Topalel, Ayhan Kaydu, Cem K. Kaçar, Erhan Gökçek, Enes Sırma, Ömer Erdağ, Sedat Kaya

**Affiliations:** 1 Department of Anesthesia and Reanimation, Health Science University Gazi Yaşargil Training and Research Hospital, Diyarbakır 21070, Turkey; osmanuzundere@gmail.com (O.U.); acilfatma@gmail.com (F.A.); selentopalel26@gmail.com (S.T.); cem.kacar@hotmail.com (C.K.K.); sedat1966@hotmail.com (S.K.); 2 Department of Child and Adolescent Psychiatry, Health Science University, Gazi Yaşargil Education and Research Hospital, Diyarbakır 21070, Turkey; suleyman.kizildag7@gmail.com; 3 Department of Anesthesiology and Reanimation, Faculty of Medicine, Dicle University, Diyarbakır 21090, Turkey; merdalerbatur@gmail.com (M.E.E.); akaydu@hotmail.com (A.K.); gokcekerhan_44@hotmail.com (E.G.); 4 Department of Autorhynopharyngeology, Health Science University, Gazi Yaşargil Education and Research Hospital, Diyarbakır 21070, Turkey; enessirmamd@gmail.com (E.S.); omererdagg@gmail.com (Ö.E.)

**Keywords:** adenotonsillectomy, anxiety, preoperative visual explanation

## Abstract

**Background/Objectives**: This study aimed to investigate the effects of explaining the perioperative process to pediatric patients scheduled for adenotonsillectomy using pictures on their anxiety levels. **Methods**: A prospective, randomized controlled trial was conducted, enrolling 58 patients. The patients were divided into two groups: Group 1 (*n* = 29), where the perioperative process was explained using pictures, and Group 2 (*n* = 29), the control group, where no pictures were used. Child anxiety was assessed using the modified Yale Preoperative Anxiety Scale Short Form (mYPAS-SF) at five observation time points before anesthesia induction. Parents’ anxiety was measured using the Visual Analog Scale for Anxiety. **Results**: Patients in Group 1 had significantly lower heart rates during induction and the intraoperative period compared to Group 2 (*p* = 0.031, *p* = 0.025, respectively). In terms of anxiety and RSAS scores, patients in Group 1 had significantly lower mYPAS-SF scores at time points t2, t3, t4, and t5 compared to Group 2 (t2: *p* = 0.001; t3–t5: *p* < 0.001). No significant difference was observed at t1 (*p* = 0.068). The mean RSAS scores were also significantly lower in Group 1 (*p* = 0.029). Parents’ anxiety was significantly lower in Group 1 at all three time points (t1: *p* = 0.017; t2: *p* = 0.006; t3: *p* = 0.036). **Conclusions**: Our study results demonstrate that illustrating the perioperative process to children undergoing adenotonsillectomy can significantly reduce preoperative anxiety and prevent awakening agitation. Given its ease of implementation, we believe that using visual aids to explain the perioperative process to pediatric patients can facilitate process management for patients, parents, and physicians.

## 1. Introduction

Anxiety is a negative emotional state triggered by stressful or threatening conditions [1]. Approximately 60% of children experience significant anxiety before surgery. These children may exhibit distress behaviors such as fear, crying, clinging to loved ones, or attempting to escape, which can complicate anesthesia induction [2]. High preoperative anxiety levels are associated with difficulties during anesthesia induction, increased postoperative analgesic requirements, emergence agitation, and negative behavioral changes after surgery [3,4].

Sedative premedication is one of the most commonly used interventions to prevent and treat preoperative anxiety in children [4,5]. However, sedatives are typically administered 30 min before surgery, while children’s anxiety often begins at hospital admission or even earlier. Additionally, sedatives are associated with potential adverse effects [6]. Non-pharmacological alternatives, such as psychological preparation programs [7] and parental presence [8] during anesthesia induction, have been proposed.

The primary aim of this study was to test the hypothesis that explaining the perioperative process using pictures would reduce anxiety levels in children undergoing adenotonsillectomy. The secondary aim was to investigate the effects of this intervention on emergence agitation, hemodynamic parameters, and parental anxiety.

## 2. Materials and Methods

### 2.1. Study Design, Population, and Data

The research adhered to the CONSORT guidelines. This study was designed as a prospective, randomized, and controlled study and commenced after obtaining ethical approval from the committee at Gazi Yaşargil Training and Research Hospital, under decision number 43 dated 10 May 2024. The trial was registered with the Clinical Trials Registry (NCT06427928) on 16 May 2024. Patient enrollment began after trial registration, and both verbal and written informed consent were obtained from all participants before their inclusion in the study. The study was completed on 27 January 2025. The study was meticulously planned following the ethical guidelines outlined in the 2013 Declaration of Helsinki. Informed consent for participation was obtained from the parent or legal guardian of each participant under 16. This study adheres to CONSORT guidelines.

### 2.2. Eligibility Criteria

**Figure 1:**
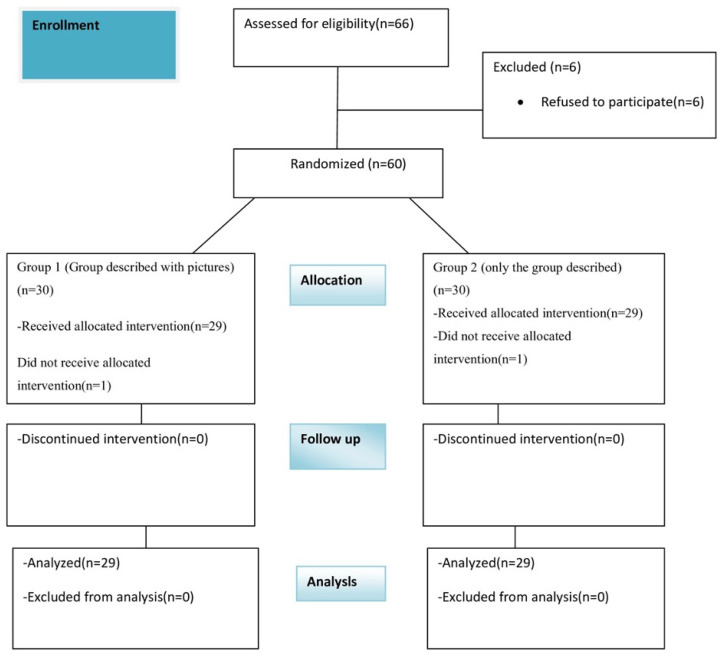
Patient enrollment flowchart.

Children aged 5 to 12 years [9,10], with ASA physical status I, who did not have any anxiety problems and underwent elective adenotonsillectomy under general anesthesia were included in the study. The patient enrollment flowchart is shown in Figure 1. Exclusion criteria included parents’ rejection, significant hearing or vision impairments, developmental delay, neurological diseases, and having had any previous surgery.

### 2.3. Randomization

Patients were randomly allocated to either Group 1 or Group 2 through a computerized random number generator, and the assignments were secured within sealed and numbered envelopes.

Group 1: Patients who received information about the perioperative process in addition to traditional information provided through pictures.

Group 2: Patients who received traditional information about the perioperative process without pictures.

### 2.4. PICO Strategy

Participants/Population(s): Both male and female patients between the ages of 5 and 12 who will undergo adenotonsillectomy.

Intervention: Patients who received information about the perioperative process in addition to traditional information provided through pictures.

Control: Patients who received traditional information about the perioperative process without pictures.

Outcomes: modified Yale Preoperative Anxiety Scale Short Form (mYPAS-SF), Visual Analogue Scale of parents (VAS), Riker Sedation–Agitation Scale (RSAS).

### 2.5. Masking

Research data collectors, anesthesiologists, surgeons, and operating room nurses were unaware of group assignment. General information, including fasting duration, surgical procedure, anesthesia risks, and pain management, was provided to both groups during their first visit to the anesthesia clinic before surgery.

**Figure 2:**
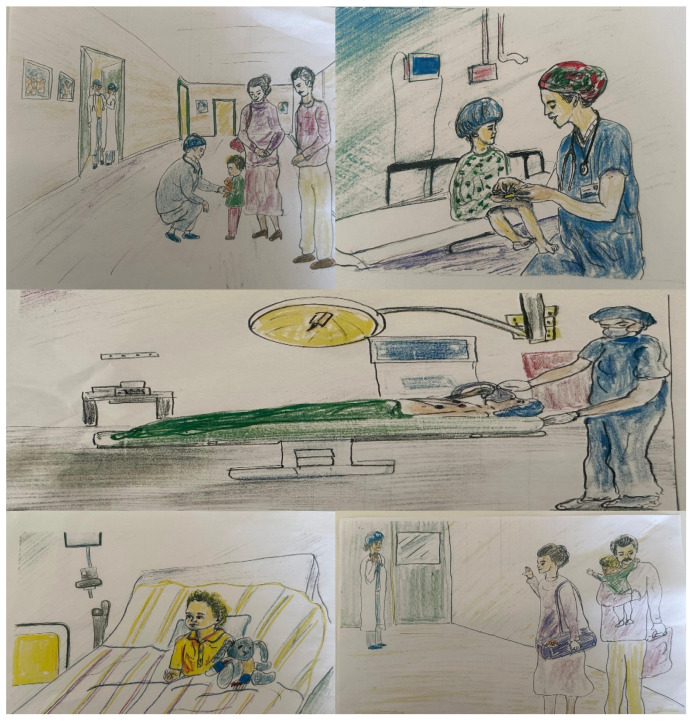
Pictures drawn for visual explanation.

Patients were admitted to the surgical ward the day before surgery and were assessed by an anesthesiologist from the research team during the pre-anesthesia visit. This allowed parents and children to be given more details about the surgery and anesthesia and to have their concerns addressed and questions answered. In this study, two anesthesiologists were assigned to inform and evaluate the patients. One provided the information, while the other blindly assessed the anxiety and other parameters of the patients and their parents after the information was provided. The perioperative process was explained to the patients in Group 1 using pictures drawn by a child psychiatrist (Figure 2), while the same anesthesiologist explained the process to the patients in Group 2 using traditional verbal instructions.

For anesthesia induction and patient compliance, all patients arrived at the preoperative waiting area approximately 30 min before the start of anesthesia, and no premedication was given. In our hospital, one parent was allowed to accompany the child to the waiting area, and the parent was separated from the child just before entering the operating room.

**Figure 3:**
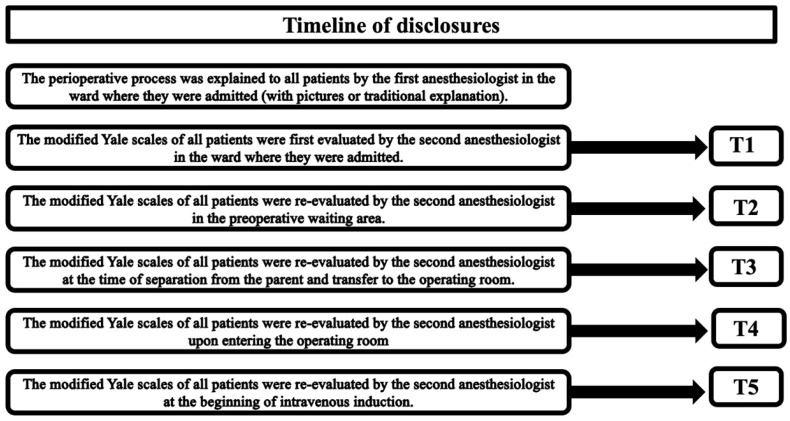
Timeline of disclosures.

The modified Yale Preoperative Anxiety Scale Short Form (mYPAS-SF) was used by a blind anesthesiologist to assess all children’s anxiety [11]. Children’s anxiety was assessed as follows: during the preanesthetic visit by the anesthesiologist shortly after admission to the surgical ward (T1), in the preoperative waiting area (T2), at the time of separation from the parent to the operating room (T3), upon entry to the operating room (T4), and at the time of starting intravenous induction (T5) (Figure 3).

Patients’ heart rates were recorded before induction, after intubation, and finally after extubation.

**Figure 4:**
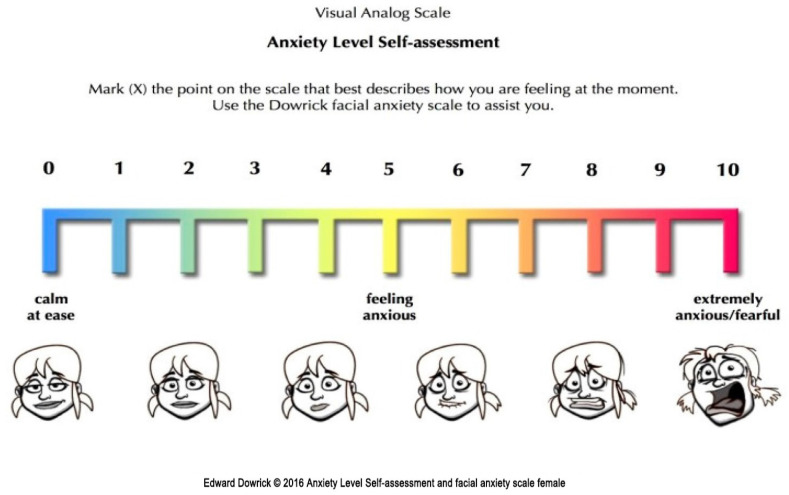
VAS-A System for evaluating parents’ anxiety [12].

Parental anxiety was measured using a visual analog scale (VAS) from T1 to T3 [12]. surgical ward (T1), in the preoperative waiting area (T2), and at the time of separation from their child (T3) (Figure 3). The scale ranged from 0 to 10, where 0 represented no anxiety, and 10 represented extreme anxiety (Figure 4).

### 2.6. Routine Preoperative and Intraoperative Procedures

No patients received premedication. All patients began standard anesthesia monitoring, including electrocardiography, noninvasive blood pressure, peripheral oxygen saturation, and temperature assessment on the operating table. All patients received preoxygenation at a rate of 4 L/min for 3 min before the induction of anesthesia. To induce anesthesia, 1 µg/kg fentanyl (Fentaver 0.5 mg/10 mL, Haver Farma İlaç A.Ş., Osel İlaç San. Ve Tic. A.Ş., İstanbul, Türkiye) and 2 mg/kg propofol (Propofol-PF 1%, Polifarma İlaç Sanayi ve Ticaret A.Ş., Tekirdağ, Türkiye) were administered intravenously. Additionally, 0.6 mg/kg rocuronium (Muscobloc 50 mg/5 mL, Polifarma İlaç Sanayi ve Ticaret A.Ş., Tekirdağ, Türkiye) was administered intravenously within 20–30 s to induce neuromuscular blockade. After the administration of medications, all patients were manually ventilated with 100% oxygen until endotracheal intubation was accomplished. Endotracheal intubation was performed using a cuffed endotracheal tube of suitable diameter corresponding to the patient’s age and body structure. Following endotracheal intubation, ventilation was conducted in volume control mode using the Dräger Primus (Drägerwerk AG & Co. KGaA, Lübeck, Germany) anesthesia device, with a tidal volume of 6–8 mL/kg, respiratory frequency set at 12–18/min, and positive end-expiratory pressure maintained at 3–5 cmH_2_O. Soda-lime (SorboLime, Berkim Kimya San. ve Tic. Ltd. Şti., Ankara, Turkey) was used as the CO_2_ absorbent in the anesthesia machine. All patients received sevoflurane inhalation anesthesia according to the study’s standard protocol. Anesthesia depth was systematically monitored and recorded, adjusted for patient age, with an average minimum alveolar concentration (MAC) of 1.

Demographic data such as age, gender, and body weight, as well as heart rate (HR) were recorded before surgery, after intubation, and during extubation. Additionally, the duration of surgery and anesthesia was documented. In the postoperative period, on the operating table after extubation, the Riker Agitation Scale (RSAS) was recorded.

### 2.7. Statistical Analysis

The required sample size was calculated using G*Power software (version 3.1.9.4; University of Kiel, Kiel, Germany). A minimum of 58 patients (29 in Group 1 and 29 in the control group [group 2]) was determined, assuming a one-tailed alpha error of 0.05, a power of 0.80, an allocation ratio of N2/N1 = 1, and an effect size of 0.67 (Table 1) [13,14,15].

Statistical analysis was performed using SPSS 26.0 software for Windows (SPSS Inc., Chicago, IL, USA). The Kolmogorov–Smirnov test was used to assess the normality of the data. Continuous data are presented as mean ± standard deviation, while categorical data are expressed as frequencies and percentages. Categorical data were compared using the chi-square and Fisher’s exact tests. For normally distributed data, Student’s *t*-test was used, while the Mann–Whitney U test was applied for non-normally distributed data. A *p*-value < 0.05 was considered statistically significant. To reduce the risk of type I error inflation due to multiple testing, the Bonferroni correction was applied. Accordingly, the adjusted significance threshold was set at *p* < 0.01 (0.05/5). The effect size (r) was calculated using the formula r = Z/√ N, where Z represents the z-value obtained from the Mann–Whitney U test and N is the total sample size.

## 3. Results

A total of 66 patients were initially enrolled in the study. Six patients declined to participate, and two patients were excluded due to adverse respiratory events, resulting in 58 patients completing the study (Figure 1). The mean age of the included patients was 7.34 ± 1.36 years. Demographic data of the patients are presented in Table 2. No significant differences were observed between the two groups in terms of age, weight, gender, anesthesia duration, or surgery duration (*p* > 0.05 for each).

When comparing heart rates between the two groups, patients in Group 1 had significantly lower heart rates during induction and the intraoperative period compared to Group 2 (*p* = 0.031 and *p* = 0.025, respectively). However, no significant difference was observed in postoperative heart rates (*p* = 0.583).

In terms of anxiety and RSAS scores, patients in Group 1 had significantly lower mYPAS-SF scores at time points t2, t3, t4, and t5 compared to Group 2 (t2: *p* = 0.001; t3–t5: *p* < 0.001). No significant difference was observed at t1 (*p* = 0.068). The mean RSAS scores were also significantly lower in Group 1 compared to Group 2 (*p* = 0.029—a finding that was also associated with parental anxiety, as measured by the VAS-A (Table 3).

## 4. Discussion

In this study, we investigated the effect of explaining the perioperative process using pictures on anxiety levels in children undergoing adenotonsillectomy. We found that children who were shown pictures had significantly lower anxiety levels. Additionally, their parents also exhibited lower anxiety levels, and the children experienced a smoother emergence from anesthesia.

Traditional methods of explaining anesthesia and surgery may not fully help children understand the process or may even increase their psychological burden due to the abstract nature of the information [4]. Previous studies have explored various preoperative preparation programs, including sedative premedication, hypnosis, parental presence, behavioral preparation programs, music therapy, and acupuncture [16,17,18]. Some of these methods, such as midazolam, parental presence, and behavioral preparation programs, are commonly used in pediatric patients [19]. Currently, parental presence during anesthesia induction is a popular evidence-based behavioral intervention, and it has been confirmed that this approach not only reduces children’s anxiety but also improves their cooperation [20]. However, overly anxious parents may inadvertently increase their child’s anxiety during induction [21]. While pharmacological interventions such as sedatives are effective in alleviating children’s anxiety, they are associated with delayed discharge [22] and adverse behavioral changes [19,23]. Additionally, administering oral premedication to children can be challenging due to their reluctance or refusal. In contrast, explaining the process using pictures is easy to implement and has no side effects.

Initial studies on audiovisual distraction during anesthesia induction in pediatric patients have used technologies such as smartphones, video glasses, and even traditional laparoscopic surgical monitors. These tools have been shown to reduce mYPAS scores [24]. Yang et al. demonstrated that providing children with an animated picture book about surgery was associated with lower anxiety levels [15]. In our study, we used hand-drawn pictures to familiarize children with the operating room environment and the surgical process. To our knowledge, this is among the few studies that utilize hand-drawn pictures created by a child psychiatrist in a randomized controlled design. Given the increasing use of technology today, this approach is beneficial in settings with limited access to advanced technology. Indeed, Bedaso et al. highlighted that in low- and middle-income countries, one in two patients experiences preoperative anxiety, underscoring the need for accessible interventions [25].

Cordray et al. conducted a prospective randomized controlled trial in which children aged 5–12 years were divided into two groups: one group received a pop-up book about anesthesia, while the other received standard information. The group that received the book showed a significant reduction in anxiety during anesthesia induction [10]. Similarly, Lee et al. found that showing animated cartoons to children aged 3–7 years before induction reduced preoperative anxiety [26]. Hou et al. also reported lower anxiety levels in children who received preoperative education using comic books, videos, and coloring books [27]. In contrast, Kulkarni et al. found no significant difference in anxiety levels between children who received a comic brochure and those who received only verbal information [28]. Our findings align with most studies in the literature and support the use of visual presentation techniques to reduce preoperative anxiety in pediatric patients. Our study contributes to the existing literature by demonstrating that explaining the perioperative process with simple visuals can significantly reduce both physiological (e.g., heart rate) and behavioral indicators of anxiety in children.

It is well-established that children’s preoperative anxiety positively correlates with parental anxiety. Li et al. observed that parental anxiety can make children feel more fearful and less cooperative [29]. Wolfer and Visintainer emphasized that parental stress is a significant factor in determining how a child emotionally responds to the stress of surgery [30]. They also provided evidence that parental stress related to upcoming surgery can easily be indirectly transferred to the child, potentially harming their recovery. Therefore, efforts should be made to reduce parental stress through behavioral or other interventions that can also alleviate children’s anxiety. Hou et al. found that parents whose children received preoperative education using visual aids had lower anxiety levels [28]. This finding suggests that interventions that not only support the child’s psychological preparedness but also reduce the parent’s emotional burden can provide twofold benefits. Visual-based informational methods should be considered an important tool for family-centered care approaches and should be more widely implemented in clinical practice. Furthermore, future research should thoroughly examine how the indirect effects of parental anxiety translate into broader domains such as surgical outcomes, patient satisfaction, and healthcare utilization. Therefore, developing holistic strategies focused on both the child and the parent in the preoperative period can make the pediatric surgical experience less traumatic and more positive.

### Limitations

Our study has several limitations. First, the pictures were shown to children one day before surgery and again on the day of surgery, which may not have fully addressed preoperative anxiety. Second, there was no standardized scale or scoring system to assess how deeply the children understood the pictures. Third, the educational background of the parents was not evaluated. Fourth, the visual materials used are not standard or accepted. Therefore, it is not possible to standardize our method. Our study was designed for a single center, a single operation, and a limited population. Therefore, our results are not generalizable. Our results may not apply to ASA II-III-IV patients. We only included adenotonsillectomy patients in our study. Although the images used in our study were drawn by a child psychiatrist, it seems impossible to standardize these images. In this study, all children, regardless of age, were presented with the same visual model. Considering that cognitive awareness levels can vary according to age, this may constitute a methodological limitation. Additionally, because patients and their parents were informed about the study design, patients in group 1 may have believed they were receiving enhanced preparation. Although it was not possible to physically blind the participants and their parents due to the nature of the intervention, each group was exposed only to the form of preoperative information assigned to them and was unaware of the existence of an alternative method. Therefore, potential expectation or placebo effects were likely minimized. Based on this design, the study was conducted under a double-blind structure in terms of both group assignment and outcome assessment. This may have caused the patients to feel more confident due to a placebo effect, which may have influenced the study results. Finally, postoperative anxiety levels were not assessed.

## 5. Conclusions

Our study results demonstrate that illustrating the perioperative process in children undergoing adenotonsillectomy can significantly reduce preoperative anxiety and prevent awakening agitation. Given its ease of implementation, we believe that using visual aids to explain the perioperative process to pediatric patients can facilitate process management for patients, parents, and physicians.

**KEY MESSAGES**

**WHAT IS KNOWN?**

- Preoperative anxiety is a common issue in children, with approximately 60% experiencing significant anxiety before surgery, which can lead to distress behaviors and complications during anesthesia induction.
- Traditional methods like sedative premedication and parental presence during induction are commonly used to reduce preoperative anxiety, but they have limitations, including delayed discharge and potential adverse effects.

**WHAT IS NEW?**

- This study introduces a novel approach using hand-drawn pictures to explain the perioperative process to children, significantly reducing their preoperative anxiety and emergence agitation.
- The findings demonstrate that visual aids not only lower children’s anxiety but also reduce parental anxiety, highlighting the interconnectedness of child and parent stress levels in the surgical context.

The study provides evidence that simple, low-cost visual interventions can be effective in reducing preoperative anxiety, particularly in settings with limited access to advanced technology.

## Figures and Tables

**Table 1:** Power Analysis Summary Table.

We Performed the POWER Analysis [13]:	Before Starting
On the primary outcome:	The modified Yale Preoperative Anxiety Scale Short Form (mYPAS-SF) [14]
Based on the two-tailed statistical test:	One-tailed
And accepting the cutoff for significance (α):	0.05
and a power (1–β) of:	0.80
The variability of the primary outcome was:	In the study by Yang et al., mYPAS-SF scores were taken into account (group P mean 51.9 ± 23.6, group C mean 67.2 ± 22) [15]
We considered as clinically relevant a difference (or a different effect, please specify) of:	1
Consequently, the effect size was:	0.67
The total sample size needed was:	58

**Table 2:** Baseline characteristics of the total population.

Parameters	Visual Information Group 1 (*n* = 29)	Standard Information Group 2 (*n* = 29)	Total Population (*n* = 58)	*p*-Value
Age	7.48 ± 1.18	7.21 ± 1.52	7.34 ± 1.36	0.444
Female gender, *n* (%)	15 (51.7)	15 (51.7)	30 (51.7)	1.000
Anesthesia time (min)	40.7 ± 6.1	39.7 ± 8.3	40.2 ± 7.3	0.592
Surgery time (min)	35.5 ± 5.9	34 ± 8.4	34.7 ± 7.2	0.418
Weight (kg)	25.9 ± 6.1	23.9 ± 5.7	24.9 ± 5.9	0.210
Heart rate-1 (bpm) *	112.4 ± 10.4	106.1 ± 11.2	109.3 ± 11.2	0.031
Heart rate-2 (bpm) **	123.4 ± 11.4	116.1 ± 12.8	119.8 ± 12.6	0.025
Heart rate-3 (bpm) ***	118.5 ± 10.1	117 ± 10.3	117.8 ± 10.2	0.583

*; before induction, **; after intubation, ***; after extubation, bpm; beat per minute.

**Table 3:** Modified Yale preoperative anxiety scale—short form of children and visual analog scale of parents.

Parameters	Visual Information (Group 1) (*n* = 29)	Standard Information (Group 2) (*n* = 29)	Total Population (*n* = 58)	Effect Size (r)	*p*-Value	Bonferroni α = 0.01
**RSAS**	4.17 ± 0.47	4.66 ± 1.05	4.41 ± 0.84	0.29	**0.029**	
**mYPAS-SF-1**	39.36 ± 9.6	43.59 ± 7.9	41.45 ± 8.9	0.24	0.068	NS
**mYPAS-SF-2**	41.86 ± 8.1	50.32 ± 10.7	46.09 ± 10.4	0.43	**0.001**	**Significant**
**mYPAS-SF-3**	42.32 ± 7	54.86 ± 13.1	48.59 ± 12.1	0.46	**<0.001**	**Significant**
**mYPAS-SF-4**	43.73 ± 7.9	56.59 ± 13.6	50.14 ± 12.8	0.46	**<0.001**	**Significant**
**mYPAS-SF-5**	44.82 ± 8.1	58.77 ± 13.6	51.82 ± 13.1	0.46	**<0.001**	**Significant**
**VAS-A 1 (parents)**	3.86 ± 0.92	4.59 ± 1.3	4.22 ± 1.17	0.44	**0.017**	NS
**VAS-A 2 (parents)**	4.55 ± 1.1	5.41 ± 1.24	4.98 ± 1.22	0.51	**0.006**	**Significant**
**VAS-A 3 (parents)**	5.21 ± 1.11	5.93 ± 1.43	5.57 ± 1.33	0.39	**0.036**	NS

RSAS: Riker sedation agitation scale, mYPAS-SF: Modified Yale Preoperative Anxiety Scale—Short Form, VAS: Visual analog scale, NS: Non-significant. Statistically significant parameters are highlighted in bold.

## Data Availability

The data associated with the paper are not publicly available but are available from the corresponding author on reasonable request.
